# Translation of therapeutic strategies to modulate B cell reponses from non-human primate models to human kidney transplantation

**DOI:** 10.3389/frtra.2023.1176796

**Published:** 2023-04-19

**Authors:** Stuart Knechtle, Jean Kwun, Shengli Song, Annette Jackson, Kitza Williams, Scott Sanoff

**Affiliations:** ^1^ Duke Transplant Center, Duke University Medical Center, Durham, NC, United States; ^2^ Department of Surgery, Duke University, Durham, NC, United States; ^3^ Department of Medicine, Duke University Hospital, Durham NC, United States

**Keywords:** kidney, transplant, antibody, rejection, desensitization

## Abstract

Using novel drugs targeting lymphocyte costimulation, cytokines, antibody, complement, and plasma cells, we have developed strategies in a non-human primate model to modulate the B cell response to incompatible kidney transplants. After more than two decades of research supported by mechanistic studies, this has resulted in clinically relevant approaches that are currently enrolling in clinical trials or preparing for such. In this manner, we aim to address the problems of HLA sensitization for very highly sensitized patients awaiting transplantation and the unmet need of effective treatment for antibody-mediated rejection.

## 1. Background and introduction

The B cell response to solid organ transplants remains the leading cause of immunologic graft loss and an unsolved problem with respect to therapeutic strategies of prevention and treatment (1). We have approached this immunologic challenge using animal models to evaluate interventions that target B cells, plasma cells, cytokines, T-B cell interactions, antibodies, and complement with varying degrees of efficacy and safety. The principal animal model we have employed has been a non-human primate (NHP) renal allograft model due to the genetic similarities of rhesus monkeys and humans, the same organ transplant type as is most commonly transplanted in humans, and high compatibility of candidate drugs designed for human use with this model. The availability of molecular typing of NHP major histocompatibility complex, support of the work by NIH funding, and available veterinarian support for such work has allowed considerable progress that has led to initiation of clinical trials in humans who would otherwise have little access to kidney transplantation or poor prognosis due to B cell mediated rejection (2).Work in this area has been greatly aided by the progress made in different but related scientific and therapeutic areas such as multiple myeloma, costimulation blockade, antibody and complement research, cytokine blockade, and basic B cell and plasma cell research. The proliferation of new drugs, some FDA-approved, has allowed us to investigate multi-drug regimens in a university research context where strategies are not limited by loyalty to or ownership of a particular product. We aim to not only develop better treatments for patients but to base these on an improved mechanistic understanding of B cell immunology; hence, our collaboration with an extensive team of investigators has enhanced our learning. Lessons learned in the allo-immunized transplant recipient appear to also apply to the immune response to pig-to-primate kidney xenotransplants, allowing extension of the methods and strategies from allogeneic models to the evolving field of xenotransplantation as well.

## 2. Preclinical observations

Success in controlling the T cell-mediated response to organ transplants improves graft and patient survival dramatically but has not adequately addressed the transplant humoral response. Additionally, the lack of adequate means to manage the humoral response (e.g., B cell, plasma cell, antibody, or complement) has contributed to long-term organ transplant outcomes being only marginally improved over the past few decades (3). The impact of humoral mechanisms on transplant rejection are more accentuated in sensitized patients who pre- or post-transplant develop HLA antibodies due to a sensitizing exposure to donor HLA antigen and through inadequate suppression of the humoral response. Fortunately, we now have a better armamentarium to target these B cell responses, and in many cases these drugs were developed to treat diseases in neighboring non-transplant fields such as multiple myeloma. In order to select promising candidates for clinical translation in transplantation, we developed a nonhuman primate (NHP) sensitized model that encompasses both sensitizing exposure and clinically-relevant immunosuppression.

### 2.1. Sensitized nonhuman primate model

Our sensitized model involves two sequential skin grafts from maximally MHC mismatched donor monkeys (4–6). Serial skin grafting reliably leads to alloantibody production as confirmed by flow crossmatch and accelerated rejection of the second skin graft. Following sensitization, NHPs receive desensitization therapy for one month and subsequently undergo renal allotransplants with the kidney from the same skin donor. The renal transplants are life-sustaining as native kidneys are both removed. The immune response of the monkey is then assessed post-operatively. We have established a baseline sensitized control group treated with conventional post-transplant immunosuppression (tacrolimus/MMF/steroid). Sensitized animals show pronounced AMR, as evidenced by thrombotic microangiopathy (TMA) with discrete thrombi in glomeruli, glomerulitis, and pertitubular capilaritis accompanied by mild interstitial inflammation after kidney transplantation (4, 7), changes that reflect the same pathology seen in analogous human renal transplants. Therefore, the model provides a basic platform to evaluate clinically relevant post-transplant immunosuppressive regimens for sensitized human transplant recipients. We acknowledge that differences exist between immune responses of different species, but the NHP model is the closest available animal model in which to conduct such research and has many advantages including the ability to use agents developed for human use, as most have similar biologic activity in rhesus monkey (8).

### 2.2. Desensitization with costimulation blockade (CoB) and proteasome inhibitor (PI) in NHP model

We have evaluated various pharmacological desensitization approaches in our sensitized NHP model (summarized in Table 1), which includes CoB [belatacept (CTLA4-Ig) (5, 9, 10); lulizumab (anti-CD28mAb) (13)], tocilizumab (anti-IL6R), plerixafor (anti-CXCR4) (6), tabalumab (anti-BAFF), proteasome inhibitors (bortezomib and carfilzomib) (15), daratumumab (anti-CD38mAb) (6), rozanolixizumab (anti-FcRn mAb)(14), and complement inhibitor (anti-C3) (11). We show that targeting a single pathway does not readily achieve durable desensitization. For example, solely targeting PC with bortezomib or solely targeting the germinal center (GC) response with CoB failed to decrease serum DSA level (9, 15). CoB did not affect preformed PCs while PC depletion was rapidly compensated by the GC response (15). Targeting antibody by interfering with IgG recycling *via* FcRn showed a significant reduction of preformed DSA but did not prevent or reduce post-transplant DSA following an anamnestic response (14). Instead of targeting a single pathway, we targeted both T cell help for the B cells and plasma cells with the combination of CoB and PI which reliably reduced preformed DSA and significantly prolonged graft survival in sensitized NHP. We reported that animals treated with carfilzomib and belatacept showed significantly reduced early AMR and prolonged graft survival (10). Despite these encouraging outcomes, some animals developed DSA and late AMR gradually**.** This may reflect the limitations of the current CNI-based immunosuppressive regimen (Tacrolimus/MMF/Steroid) with respect to controlling the post-transplant humoral response.

**Table 1:** Desensitization strategies tested in NHP preclinical model(s).

Desensitization agents	*N*	Induction	Maintenance IS	Graft Survival (days)	Outcome/ Diagnosis	Target	Reference
No treatment	3	Basiliximab	Tacrolimus/MMF/Steroid	4.67	ABMR/TCMR	N/A	(4, 9)
	7	CD4/CD8 mAb	Tacrolimus/MMF/Steroid	22	ABMR	N/A	(4, 5)
	4	CD4/CD8mAb	Tacrolimus/MMF/Steroid	5	ABMR	N/A	(6, 10–12)
	5	rhATG	Tacrolimus/MMF/Steroid	5.8	ABMR/TCMR	N/A	(13, 14)
Belatacept	3	N/A	N/A	N/A	No DSA reduction	GC response	(9)
Thrombalexin	3	CD4/CD8 mAb	Tacrolimus/MMF/Steroid	6.6	ABMR	TMA	(7)
Belatacept + 2C10	3	N/A	N/A	N/A	No DSA reduction	GC response	(9)
Bortezomib	4	N/A	N/A	N/A	No DSA reduction	Plasma Cells	(15)
Belatacept + 2C10 + Bortezomib	3	Basiliximab	Tacrolimus/MMF/Steroid	>58.6	No ABMR, Weight loss	Plasma Cells + GC response	(9)
	5	CD4/CD8 mAbs	Tacrolimus/MMF/Steroid	>40	No ABMR, viral complications	Plasma Cells + GC response	(5)
Daratumumab + Plerixafor	4	CD4/CD8 mAb	Tacrolimus/MMF/Steroid	21.6	TCMR/ABMR	Plasma cell/PC niche	(6)
Carfilzomib	3	CD4/CD8 mAb	Tacrolimus/MMF/Steroid	6	ABMR	Plasma cells	(12)
Lulizumab + Carfilzomib	5	rhATG	Tacrolimus/MMF/Steroid	64.8	ABMR	Plasma cells + GC response	(13)
Rozanolixizumab (Anti-FcRn mAb)	2	rhATG	Tacrolimus/MMF/Steroid	7 and 9	ABMR	IgG	(14)
Tocilizumab + Carfilzomib	4	CD4/CD8 mAb	Tacrolimus/MMF/Steroid	24	ABMR	Plasma cells and IL-6	(12)
Compstatin (anti-C3)	6	rhATG	Tacrolimus/MMF/Steroid	22.5	ABMR	Complement C3	(11)
Belatacept + Carfilzomib	5	CD4/CD8 mAb	Tacrolimus/MMF/Steroid	>114	ABMR	Plasma cells + GC response	(10)
	5	rhATG	Tacrolimus/MMF/Steroid + Belatacept	>169	PTLD	Plasma cell + GC response	(16)
	5	rhATG	Rapamycin/MMF/Steroid + Belatacept	>92	TCMR, no ABMR	Plasma cell + GC response	(16)

ABMR, antibody-mediated rejection; TCMR, T cell mediated rejection; rhATG, rhesus anti-thymocyte globulin.

## 3. Targeting plasma cells

Plasma cell depletion using proteosome inhibitors such as bortezomib and carfilzomib, or by targeting with mAb such as daratumumab, can effectively deplete plasma cells and thereby lower alloantibody levels over time (6, 17, 18). The impact is non-specific with respect to antibodies and therefore potentially reduces protective antibodies as well as anti-HLA antibodies. Furthermore, while antibody levels can be acutely lowered by plasma cell depletion, they rebound promptly without additional intervention to suppress upstream B cell activation. We have reported several approaches to prevent rebound as summarized above in our NHP experiments. These strategies are being implemented now in human clinical trials of kidney transplantation summarized below and supported by not only the NHP data but also by small pilot studies and case reports in human transplantation (19, 20).

## 4. Measuring allo-specific B memory cells

The CTLA4-Ig costimulatory blockade agents exert both direct and indirect effects on different steps of the B cell-mediated responses. *In vitro* studies showed that, in a T cell-independent manner, Belatacept reduces plasmablast differentiation and Ig production, and downregulates the expression of Blimp-1, the master transcription factor in plasma cell differentiation (21). In the presence of CD40l and IL-21 stimulation, belatacept (21) or abatacept (22) treatment reduces expression of CD80 and CD86 on activated B cells. Belatacept also augments STAT3 phosphorylation in cultured B cells (21). As expected, the reduced expression plus blockade of CD80 and CD86 on B cells disrupts the B-Tfh cell interaction, which dampens the function of Tfh cells as evident by decreased expression of ICOS and PD1, and in turn lowers plasmablast differentiation (21).

In view of the multifaceted role of CTLA4-Ig costimulatory blockade on B cells and that both historic and *de novo* allo-responses can give rise to allo-specific memory B (Bmem) cells (23–26), the identification and tracking of allo-specific Bmem cells at clonal basis would provide more comprehensive information than serum tests do at polyclonal level for the understanding of the mechanisms and further harnessing of this immunosuppression regimen. Based on a high-throughput single B-cell culture method (27), we successfully identified allo-specific Bmem cells from sensitized patients in a preliminary study (28). We also established a multiplex reporter cell assay (28, 29) using single HLA-antigen expressing reporter cells mimicking single-antigen beads. This cost-efficient assay allows us to screen B cell culture supernatants for HLA antigen binding activities in a high-throughput manner. Ongoing efforts are being made to increase the specificity of HLA antigen probes for efficient isolation of allo-specific Bmem cells.

## 5. Assessing the impact of alloantibody

The crossmatch test hails as the gold standard in histocompatibility testing to assess alloantibody levels that could result in hyperacute or accelerated antibody mediated rejection. Crossmatch tests measure alloantibodies bound to HLA expressed on donor lymphocytes referred to as DSA, donor specific antibody (DSA) (30). The complement dependent cytotoxicity crossmatch detects higher levels of DSA capable of eliciting cell lysis while the more sensitive flow cytometric crossmatch measures lower levels of DSA bound to donor cells using an anti-IgG fluorescently conjugated antibody. Therapeutics targeting cell surface proteins (e.g., CD20, CD52) and some autoimmune conditions can interfere with crossmatch tests resulting in false positive tests.

Development of solid phase bead immunoassays for HLA-specific antibody testing has significantly improved the sensitivity and specificity for detecting DSA in the sera of transplant candidates. Current immunoassays include flow cytometric and Luminex™ -based multiplex bead assays and are constructed in three distinct forms: (1) screening beads coated with pooled HLA proteins (2) HLA phenotype or multiantigen beads using HLA proteins derived from cells and (3) single antigen beads utilizing recombinant HLA proteins (31). Bead assays allow testing of multiple sera in 96-well plates using small quantities of patient serum. Each serum is tested against 100 class I or class II beads, each bead possesses a unique fluorescence signature and is coated with a unique HLA class I or class II allele(s). Following a serum incubation, the HLA- coated beads are washed and bound HLA antibody is detected using a fluorescently labeled anti-IgG antibody. The beads are interrogated by lasers and the fluorescence from the bound anti-IgG detection antibody provides a mean fluorescence intensity (MFI) read-out that is proportional to the HLA-specific antibody bound to each particular bead. It is important to note that these assays are semi-quantitative and there are no standard curves to delineate antibody concentration (32). Testing titrated sera provides the best assessment of HLA antibody levels, however these are costly to perform given the expense of the bead regents (33).

Solid phase bead immunoassays are powerful diagnostic tools, yet have inherent limitations that must be overcome to assure analytical validity. HLA proteins, especially recombinant proteins, are prone to denaturation or misfolding leading to non-native epitopes and false positive reactions with some patient sera (34, 35). The high density of HLA proteins on the bead surface may not reflect the physiological HLA protein levels on vascular endothelium leading to an overestimation of DSA effector function and pathogenicity (36, 37). The inclusion of HLA epitope analysis and adjunct testing with HLA phenotype beads or crossmatch tests can reduce false positive and overestimations of alloantibody strength. High levels of HLA antibody bound to beads can fix complement components in sera, thereby blocking binding of the anti-IgG detection antibody leading to underestimations of HLA antibody levels (38). Pretreating sera to mitigate complement interference or testing titrated sera to remove prozone effects will provide more accurate HLA antibody measurements (39).

Current consensus guidelines to optimize HLA antibody detection and strength assessments recommend serum pretreatments, HLA pattern analysis, and the use of companion assays to confirm the presence and relative strength of HLA-specific antibodies and optimize immunological risk assessments (40, 41).

## 6. Design of human clinical trials

The initial application of combined plasma cell depletion and costimulation blockade to lower antibody levels in humans was most practical to assess in highly HLA-sensitized patients awaiting kidney transplantation. Such patients, graded by calculated panel-reactive antibody levels (cPRA), have diminished chance of receiving a kidney transplant due to pre-existing immunity to potential organ donors. Despite strategies designed to preferentially allocate donor kidneys to high PRA patients, the extremely highly sensitized (PRA > 99.9%) have little statistical chance of receiving a donor kidney with additional intervention such as desensitization. Therefore, the Immune Tolerance Network (ITN) funded a pilot study to assess the safety and efficacy of treatment with combined plasma cell depletion and costimulation blockade based on the NHP results summarized above. In fact, the ITN funded two such related trials, one based at Duke called the ADAPT trial using carfilzomib/belatacept combination therapy, and one called the ATTAIN trial based at UCSF using daratumumab (anti-CD38 mAb)/beletacept, also supported by published NHP data. These small studies were designed and begun during the COVID19 epidemic despite the limitations of clinical research during that period, and were thus subject to delays, but nevertheless took advantage of lessons learned during the epidemic about monitoring, immunization, and treatment of COVID19. While immunosuppressive drug trials are not ideally performed during an infection epidemic, we nevertheless were able to plan and initiate the trial using appropriate clinical caution. Challenges have included extrapolating from NHP to human drug dosing and schedules, establishing appropriate inclusion/exclusion criteria and endpoints, and defining appropriate safety boundaries. Both the ADAPT (NCT05017545) and ATTAIN (NCT04827979) trials are currently enrolling at the two sites without clinical safety concerns to date and await further data to allow appraisal of efficacy. Mechanistic data are being generated to inform immunologic impact of therapy and to help guide possible protocol modification.

The NIH NIAID further committed to funding a trial of combined carfilzomib/belatacept for treatment of antibody-mediated rejection of kidney transplants through its Clinical Trials in Organ Transplantation (CTOT) mechanism. (CarBel trial). This trial has completed protocol development and awaits FDA review. This work extends the NHP work showing extended graft survival in animals treated at the time of biopsy-proven AMR when treated with combined carfilzomib/belatacept (unpublished data). AMR treatment remains an unmet clinical need in transplantation in the sense that current therapies have either not been shown to be effective or have been shown to be ineffective (42). The evaluation of safety and efficacy of such novel therapies therefore offers hope for a better future for patients whose grafts develop AMR.

### 6.1. Summary of protocol of ADAPT trial

**Figure 1:**
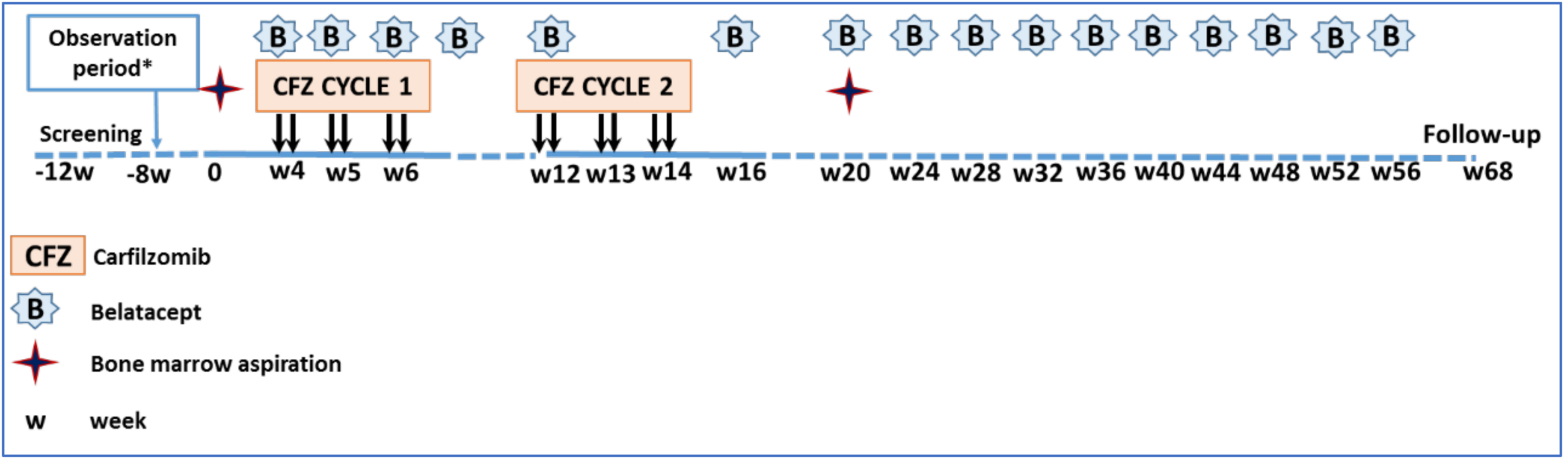
Study regimen and timeline. Each ADAPT patient serves as their own control for an initial observation period of 12 weeks to document stable alloantibody levels. Treatment begins with combined carfilzomib (CFZ) and belatacept (**B**), with the latter continuing for one year to the primary endpoing time. *8 week observation period only in cohort 1.

The ADAPT trial of desensitization seeks to test the hypothesis that a regimen of proteasome inhibition and costimulation blockade will safely and effectively reduce circulating HLA antibody and increase the likelihood of finding a compatible kidney donor in the very highly sensitized population. The study focuses on patients listed for renal transplantation who have a calculated panel reactive antibody (cPRA) of >99.9%, or cPRA > 98% and >5 years on the waitlist, or cPRA >98% with an HLA-incompatible approved living donor and not received a transplant after one year in a kidney paired exchange program. Such patients have an exceptionally low chance of receiving a kidney before they succumb to the health consequences of their renal disease. The treatment protocol for study subjects (Figure 1) includes an initial observation period to document that their cPRA remains stable, followed by combined treatment with low dose carfilzomib and standard dosing of belatacept per drug approval guidelines. The dosing strategy was based on both the NHP treatment protocol and practical needs for human clinical trial design and sampling. The clinical trial is coupled with mechanistic aims seeking to evaluate the impact of therapy on antibody levels and on allospecific memory B cells and plasma cells using novel assay systems (28, 29). In addition, the impact of treatment on ABO antibody levels will be assessed for comparison to impact on alloantibody levels. Lymph node germinal center B cell follicles will be examined by immunocytochemistry in patients who receive a kidney transplant. The influence of therapy on protective antibody vs. alloantibody will be compared. These mechanistic studies aim to shed light on both the safety and efficacy issues related to the study and to provide basic immunologic insights into memory B cells.

**Figure 2:**
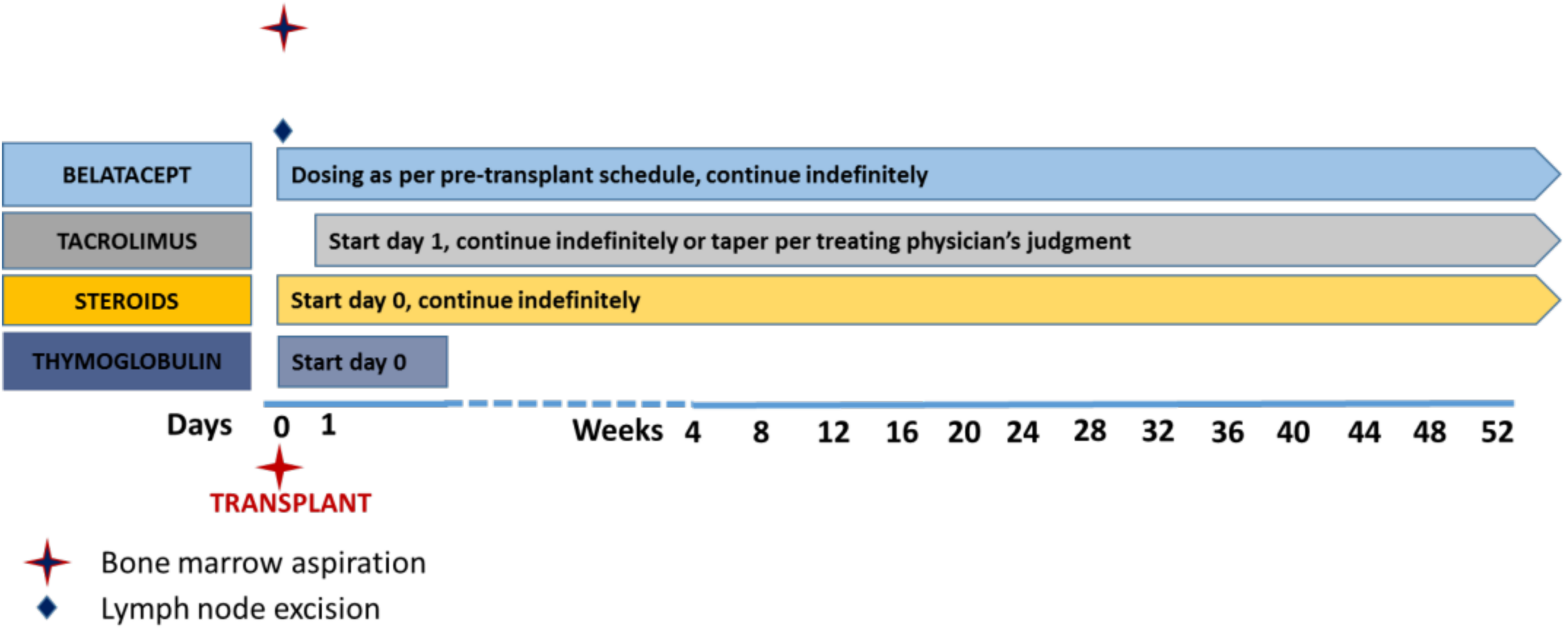
Post-transplant immunosuppression regimen. Patients in the ADAPT trial who receive transplants are induced with thymoglobulin and maintained on maintenance immunosuppression including belatacept, tacrolimus (weaned according to physician preference), and steroids. Other drugs such as MMF are per physician preference.

**Figure 3:**
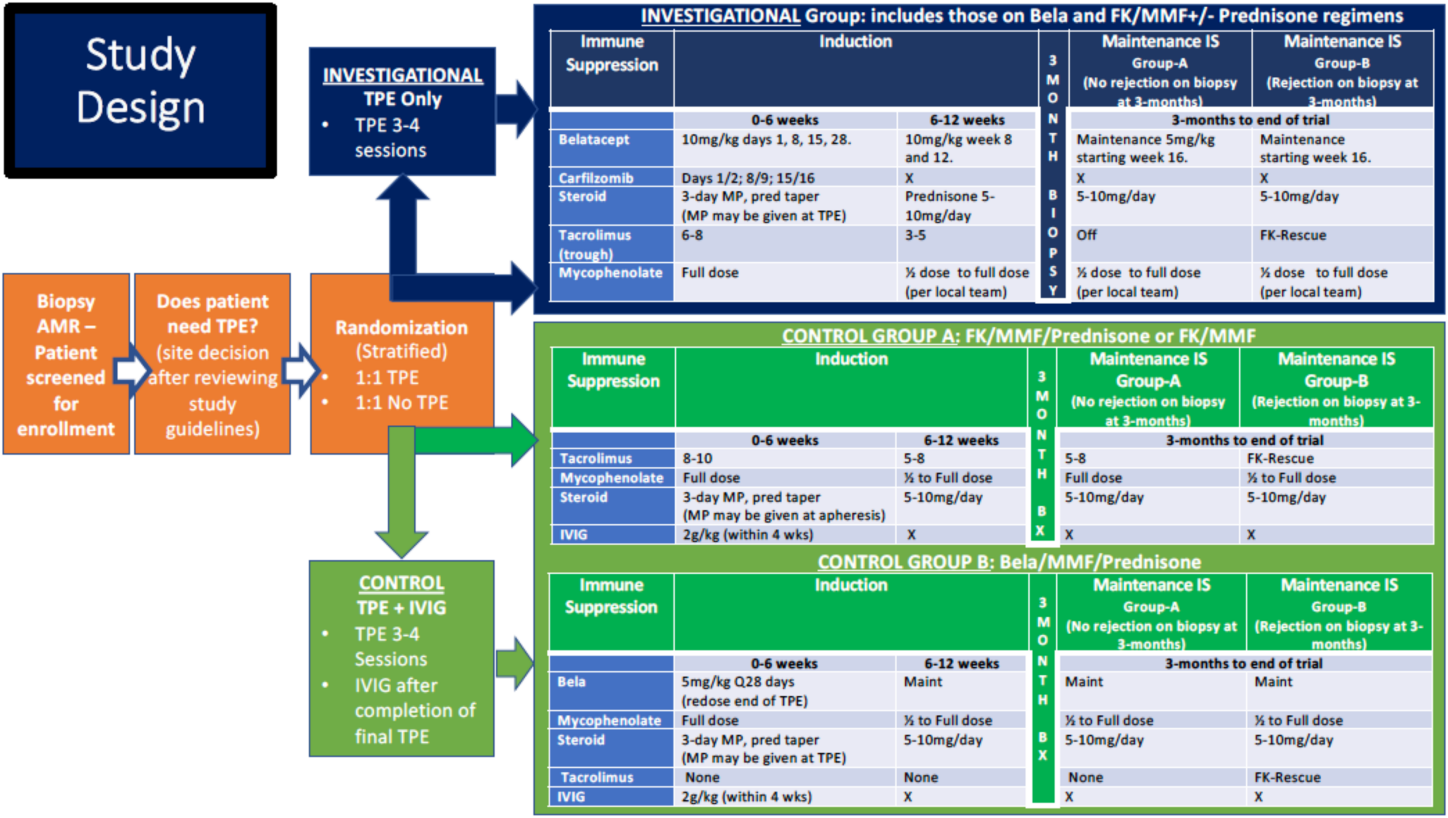
CTOT42 CarBel protocol summary.

Given the observation in NHP that ongoing belatacept after transplantation aids in suppressing AMR, human subjects who receive a kidney transplant during the study will continue on belatacept in addition to tacrolimus and steroids as ongoing immunosuppression (Figure 2). Thymoglobulin will be used as induction therapy. Both blood and bone marrow will be sampled serially for mechanistic assessment of the immune profile of patients. The primary safety endpoint of the study is the proportion of patients who remain free of grade 3 or higher infections and any malignancy; the primary efficacy endpoint is the proportion of patients who eliminate at least one HLA antibody 20 weeks after starting treatment, who have 50% or greater reduction in MFI of at least 3 HLA antibodies at 20 weeks, or who receive a kidney from a previously incompatible donor within 20 weeks without graft loss due to AMR within 4 weeks post-transplant caused by an anamnestic immune response. The trial is currently enrolling patients and at this writing is in “pause” (Figure 3). The ADAPT study is paired with a very similar study called the ATTAIN study, also ITN-funded, that is very similar in design to ADAPT but is using daratumumab (anti-CD38 mAB) in combination with belatacept to lower alloantibody levels. The study is actively enrolling currently. These two clinical trials of desensitization strategies accompanied by mechanistic assays should help inform the safety and efficacy of plasma cell targeting in conjunction with CD28 costimulation blockade.

### 6.2. Summary of CarBel trial protocol to treat antibody-mediated rejection

The strategy of dual targeting of plasma cells and costimulation blockade to prevent antibody rebound is being developed as an approach to treatment of antibody-mediated rejection (AMR) in the context of a Clinical Trials in Organ Transplantation (CTOT) trial supported by the NIH. This trial is entitled “Targeting the B Cell Response to Treat Antibody-Mediated Rejection with Carfilzomib and Belatacept (CarBel)” and the protocol is currently under FDA review. The basis of the experimental therapy (combined carfilzomib and belatacept) is based on the NHP data summarized above, the safety record of belatacept (FDA-approved for kidney transplantation), and the work of the Woodle group using carfilzomib with TPE to desensitize patients (19).

The aims of the CarBel trial are: 1. To assess the safety of CarBel therapy vs. conventional treatment of ABMR in kidney transplant subjects; and 2. To assess the efficacy of CarBel therapy vs. conventional treatment of ABMR in kidney transplant subjects as measured by improvement in slope of the Glomerular Filtration Rate (GFR) between study entry and 12 months.

The primary endpoints of the study focus on safety and efficacy and are summarized below.

#### Primary safety endpoint

Proportion of participants who do not experience any of the following from the initiation of study treatment through the end of study participation in the investigational arm compared to the conventional arm:

• meet stopping rules for safety
• grade 3 or higher infusion reaction to carfilzomib or belatacept
• grade 3 or higher infection
• malignancy

#### Primary efficacy endpoint

The primary study endpoint is the difference in estimated GFR (eGFR) slope [Chronic Kidney Disease Epidemiology Collaboration (CKD-EPI)] from enrollment to 12 months between the investigational and conventional arms.

One of the many challenges of such a study is defining a suitable control treatment since there is little evidence in support of any treatment of AMR. Commonly used treatments of AMR include optimization of maintenance immunosuppression, total plasma exchange (TPE), intravenous immune globulin (IVIg), rituximab, and proteosome inhibitors. Therefore, we chose as the control treatments either optimization of maintenance immunosuppression as this is the most common approach to chronic AMR treatment, or TPE + IVIg as this is the most commonly used treatment of acute AMR with rapidly deteriorating renal function (rise in creatinine). While we had originally intended to iBox as the primary endpoint, since the FDA has not yet approved iBox as an accepted clinical trial endpoint, we defaulted to more conventional endpoints, namely slope of GFR at one year in experimental vs. control patients. In other words, we will test the hypothesis that combined carfilzomib/belatacept therapy will preserve GFR more effectively than control treatments at one year after enrollment.

Extensive infection surveillance is included in the protocol given the known increased risk of infection associated with transplant immunosuppression. Adding additional plasma cell depletion and costimulation blockade may further increase risks of infection but an unknown risk at this time. Belatacept has been associated with increased risk of CMV infection, and carfilzomib has not been shown to be associated with increased infections in transplant patients. However, any treatment that targets immune cells may have such potential adverse effects.

We have aimed to make the inclusion criteria for the CarBel trial specific for active AMR and chronic active AMR yet focus only on AMR associated with alloantibody as our preliminary data show that the treatment does lower alloantibody levels. The study will not address AMR in the absence of alloantibody although this is now an accepted variant of AMR by Banff criteria.

We expect to learn from the CarBel trial about the safety and efficacy of the experimental therapy and, just as importantly, whether we can accurately measure the impact of the therapy on alloantibody and the frequency of allospecific B memory and plasma cells. Such assays would be of great benefit potentially in clinical immunology more broadly by informing the regulation of B cell and plasma cell responses.

## 7. Summary

We have conducted extensive preclinical testing of multiple strategies to desensitize and to treat AMR in a NHP kidney transplant model in order to develop more effective means of downregulating the B cell response to solid organ transplants that are relevant to human transplantation. The combination of plasma cell targeting and costimulation blockade safely and effectively reduces the impact of sensitization on NHP kidney transplants and therefore is being tested in analogous human clinical scenarios. The design of these trials is based on the NHP data, the feasibility of trial design in humans, and the relatively modest published data of similar approaches in humans. Given the large detrimental impact of the B cell response to transplanted organs, knowledge gained in this area is necessary and of considerable importance if we are to substantially prolong graft survival in human transplant recipients compared to current outcomes.

## Data Availability

The original contributions presented in the study are included in the article/Supplementary Material, further inquiries can be directed to the corresponding author.
